# The ubiquitin hydrolase OTUB1 promotes glioma cell stemness via suppressing ferroptosis through stabilizing SLC7A11 protein

**DOI:** 10.1080/21655979.2021.2011633

**Published:** 2021-12-19

**Authors:** Xinde Zhao, Ming Zhou, Yong Yang, Minjie Luo

**Affiliations:** Department of Pediatric Neurosurgery, Zhujiang Hospital of Southern Medical University, Guangzhou, China

**Keywords:** OTUB1, SLC7A11, stemness, glioma, ferroptosis

## Abstract

The ubiquitin hydrolase OTUB1 has been elucidated to be highly expressed in tumors, however, its roles in glioma progression are still confusing. Here, via analyzing several online datasets, OTUB1 expression was shown to be remarkably increased in glioma tissues compared to that in the adjacent tissues, and predicted a poor overall survival of glioma patients. Then OTUB1 was knocked down in glioma cells and it was found that OTUB1 knockdown significantly reduced glioma cell stemness by detecting sphere-formation ability, stemness marker expression, and ALDH activity. Mechanistic experiments revealed that OTUB1 stabilized SLC7A11 protein via directly interacting with SLC7A11, which is a key suppressor of ferripotosis. Indeed, OTUB1 knockdown triggered ferroptosis dependent on SLC7A11 expression. Notably, ectopic expression of SLC7A11 attenuated the inhibition of OTUB1 knockdown on the stemenss of glioma cells. Finally, we found a positive correlation between OTUB1 and SLC7A11 expression in clinical samples. Taken together, this work identifies a novel OTUB1/SLC7A11 axis contributing to glioma cell stemness.

## Introduction

Glioma, which occurs in the neuroectoderm, is one of the highly malignant tumors of the central nervous system [1]. The incidence rate of human glioma is very high and the prognosis of patients is usually poor [2]. Although surgical treatment and postoperative radiotherapy have made a great progress in technology, the clinical treatment effect is still disappointing in glioma. Studies show that the 5 – year mortality rate of glioma patients with higher pathological grade, like more than grade 3 in the 5th WHO classification of central nervous system tumors from 2021, is more than 95% [2]. The obvious molecular heterogeneity of glioma is considered to be a critical contributor in its treatment resistance [3]. Therefore, it is of great significance to explore the molecular mechanisms contributing to the pathological process of glioma, which can facilitate the finding of new therapeutic strategies for glioma.

Cancer stem cells (CSCs) have the ability of multi-differentiation and self-renewal, which may be the main reason of tumor occurrence, recurrence, drug resistance, and heterogeneity [4]. CSCs have been found in various tumors, such as leukemia, breast cancer, lung cancer, and glioma [5]. Targeting CSCs might be an effective method to suppress cancer occurrence, progression, and improve the therapeutic effect of drugs [6]. Protein ubiquitination is widely involved in regulating physiological and pathological processes [7]. Studies have shown that the ubiquitin proteasome system (UPS) can participate in the degradation, protein activity, and subcellular localization of most proteins in cells [8]. The ubiquitination is a reversible process and the main function of deubiquitinase is to regulate the balance of protein ubiquitination modification, and many kinds of deubiquitinases have been found. Ovarian tumor associated proteinase-1 (OTUB1) is the first confirmed-member of the deubiquitinase ovarian tumor associated proteinase family (OTUs) [9]. Studies have revealed the important effects of OTUB1 in many physiological and pathological processes, such as DNA repair and damage, apoptosis, inflammatory reaction, and tumor development [9]. Elevated OTUB1 expression has been associated with the high invasiveness, grade, and metastasis in various tumors including breast [10], lung [11], glioma [12], and ovarian [13]. Additionally, knockdown of OTUB1 can suppress glioma cell migration ability *in vitro* [12]. However, it is confusing whether OTUB1 regulates glioma stemness.

SLC7A11 belongs to be a member of the solute vector family encoding the light-chain subunit SLC7A11 (also known as xCT) of the cystine/glutamate reversal transporter X_c_^−^ system [14]. SLC7A11 is a critical suppressor of ferroptosis, which depends on iron and is a new type of programmed cell death distinct with necrosis, apoptosis, and autophagy [15]. SLC7A11 increases glutathione (GSH) synthesis by regulating glutamate release and cystine uptake, protects cells from oxidative stress and lipid peroxidation, and maintains cell redox balance, thus suppressing the ferroptotic process [14]. SLC7A11, which is overexpressed in various tumors, is closely related to glioma progression [16]. Ectopic expression of SLC7A11 has been shown to be a marker of poor glioma survival and confer chemoresistance in human glioma [17]. Additionally, SLC7A11 overexpression is associated with the stem-cell like traits in glioma [18]. Thus, it is important to elucidate the underlying mechanism by which SLC7A11 expression is regulated in glioma.

Here, we aimed to explore OTUB1 roles in glioma stemness. We firstly found that OTUB1 is significantly upregulated in glioma tissues and predicts a poor survival of glioma patients. Silencing function indicated that OTUB1 positively regulated glioma stemness. Mechanistic studies revealed that OTUB1 stabilized SLC7A11 protein via directly interacting with SLC7A11 and OTUB1 knockdown triggered ferroptosis of glioma cells. This OTUB1/SLC7A11 axis is necessary for glioma stemness. Notably, a positive correlation existed between OTUB1 and SLC7A11 expression in clinical samples. Thus, this work demonstrates that the OTUB1/SLC7A11 axis could be a potential target for glioma progression.

## Material and methods

### Clinical samples and online dataset analysis

Thirty-six pairs of glioma and adjacent tissues were obtained from Zhujiang Hospital of Southern Medical University. Each patient has approved the using of clinical samples (Supplementary Table 1). The Research Ethics Committee of Zhujiang Hospital of Southern Medical University has approved this study, which was conducted in compliance with the principles of the Declaration of Helsinki. Online datasets were downloaded from GEO (Gene Expression Omnibus) database examining gene expression in glioma and adjacent tissues, and OTUB1 expression was analyzed in these datasets (GSE100675, GSE117423, and GSE65626). Another online datasets (R2: Genomics Analysis and Visualization Platform) were used to analyze the association between OTUB1 expression and the survival of glioma patients.

### Cell culture

Glioma cell lines U373, U251, HS683, U87, T98G, SW1783, and LN-319 were purchased from Fenghui Biotechnology Co., Ltd. (Changsha, China) and cultured in DMEM medium (MeilunBio, Dalian, China) plus 10% FBS (Fetal bovine serum, Oricell, Suzhou, China) as well as 1% penicillin (Sangon, Shanghai, China) and streptomycin (Sangon) at 37°C and 5% CO_2_.

### Plasmid and siRNA construction, transfection

OTUB1 and SLC7A11 coding sequences were inserted into pcDNA 4.1 vector (pc-OTUB1 and pc-SLC7A11, respectively). SiRNAs against OTUB1 (si-OTUB1-1, 5ʹ–GAAUGCGGCCGCGAUGGCUU-3ʹ; si-OTUB1-2, 5ʹ-AUGACCAGAGCACCUCCGAC-3ʹ) and the corresponding negative control (NC, 5ʹ-GAUGGAUCCUUAGCUUAGC-3ʹ) were purchased from Biomics (Nantong, China). The transfection procedure was referred to the protocols in Lipofectamine 3000 (Thermo Fisher Scientific, Waltham, MA, USA).

### Quantitative real-time PCR (qRT-PCR)

Total RNA Extraction Kit (Cat # R1200, Solarbio, Beijing, China) was used to extract RNA from cells. One Step SuperRT-PCR Mix Kit (Cat # T2240, Solarbio) was utilized to synthesize the complementary DNA (cDNA). qRT-PCR analysis was constructed using 2× SYBR Green PCR Mastermix (Cat # SR1110, Solarbio), and GAPDH mRNA was designated as the internal reference. 2^−ΔΔct^ method was carried out to calculate the relative expression levels. The detailed primer sequences were listed as below: OTUB1 (forward: 5ʹ- ATGACCAGAGCACCTCCGACTACC-3ʹ, reverse, 5ʹ-GACCATTTACAACCACAGAAAAAC-3ʹ), SLC7A11 (forward: 5ʹ- GGTTTGTAATGATAGGGCGGCAGC-3ʹ, reverse, 5ʹ- CCATAGTAGGGACACACGGGGGAA-3ʹ), Oct4 (forward: 5ʹ- AGCGATCAAGCAGCGACTA-3ʹ, reverse, 5ʹ- GGAAAGGGACCGAGGAGTA-3ʹ), Sox2 (forward: 5ʹ- CATCACCCACAGCAAATGAC-3ʹ, reverse, 5ʹ-CAAAGCTCCTACCGTACCACT-3ʹ), CD133 (forward: 5ʹ- TGGTGGGGTATTTCTTTTGTATGT-3ʹ, reverse, 5ʹ- ACGCCTTGTCCTTGGTAGTGTTGT-3ʹ), GAPDH (forward: 5ʹ- CTTAGTTGCGTTACACCCTTTCTTG- 3ʹ, reverse, 5ʹ- CTGTCACCTTCACCGTTCCAGTTT-3ʹ).

### Western blot

The detailed procedure for protein extraction was referred to the previous study [19]. For protein stability analysis, CHX (500 nM) or MG132 (100 nM) was added into cells, respectively, and proteins from different groups were separated on the same PAGE with equal amount. Then protein was transferred to PVDF membranes. 5% nonfat milk was used to incubate with membranes at room temperature for 1 h and membranes were incubated with the primary antibody overnight at 4°C, washed with PBST for three times, incubated with the secondary antibody for 1 h, washed with PBST for three times, and finally exposed using ECL Western blotting Substrate (Cat # PE0010, Solarbio).

### Spheroid formation analysis

The detailed procedure was referred to the previous study [20]. Briefly, cells were inoculated in 24-well low-adherent cell culture plates with a density of 1000/ml, and three multiple wells were set up. Cells were cultured in sphere-culturing medium (DMEM/F12 without serum added with 1 × B27 reagent, EGF (20 ng/ml), bFGF (20 ng/ml), insulinolin (5 g/ml), hydrocortisone (1 g/ml) and 1% penicillin and streptomycin) were added to form the culture medium), and the fresh medium were added every 3 days. After 10 days of culture, the formation of spheres (>50 μm) was observed under the microscope, counted and taken photos of the microspheres in each well.

### Analysis of the levels of glutathione, cysteine, and ROS (reactive oxygen species)

The concentrations of glutathione, cysteine, and ROS were measured in glioma cells using Glutathione Colorimetric Detection Kit (Cat # K261-100, BioVision, Milpitas, CA, USA), Cysteine Assay Kit (Cat # MAK255-1KT, Sigma), and CM-H2DCF-DA (Thermo Fisher Scientific, H_2_O_2_ detection) following the standard protocols, respectively.

### Immunocoprecipitation (Co-IP)

The detailed procedure was referred to the previous work using Protein A agarose beads (50%) [21]. Briefly, before lysing, spheroids were treated with MG132 for 6 h, and then the pre-cooled RIPA buffer was used for lysing spheroids. The supernatant was obtained via centrifuging cell suspension at 4°C for 20 min by 13000 rpm.

### Ubiquitination analysis

The detailed procedure was referred to the previous work [22].

### ALDH activity detection

ALDH Activity Assay Kit (Abcam, Cambridge, MA, UK) was used to measure ALDH activity in glioma cells following the manufacturer’s protocols.

### Immunohistochemistry (IHC) assay

The detailed procedure was carried out following the protocols mentioned in the previous study [23].

### Statistical analysis

Data were denoted as the mean ± standard deviation (SD), single-factor analysis of variance (ANOVA) was used for analysis, and non-paired *t* test was used for inter-group comparison. P < 0.05 was considered to be statistically significant.

## Results

### OTUB1 is overexpressed in glioma patients and negatively correlated with the overall survival

**Figure 1.**
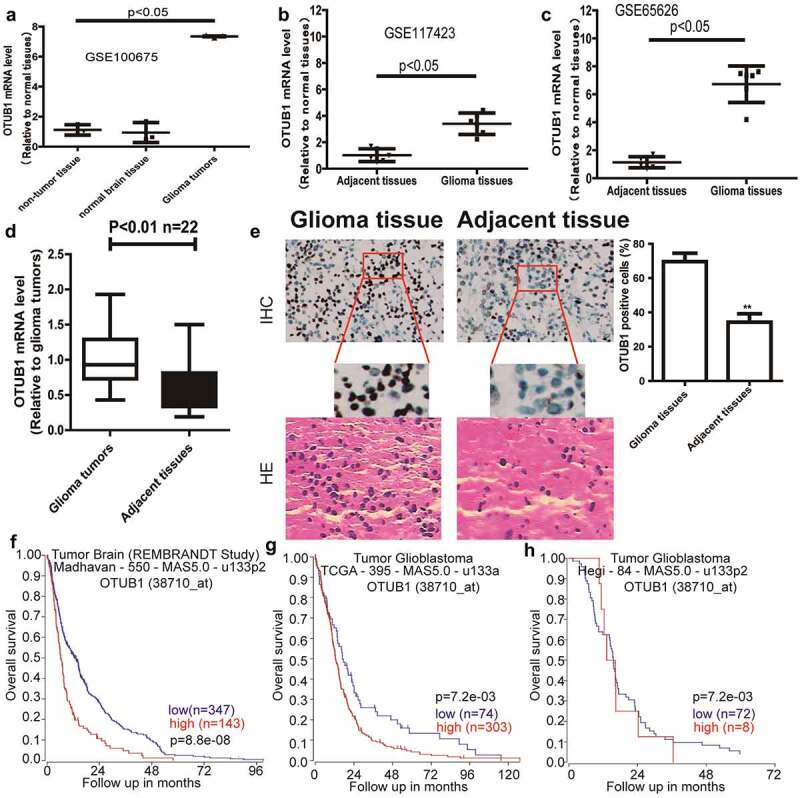
OTUB1 is overexpressed in glioma patients and predicts a poor survival. (a – c) OTUB1 expression was analyzed in GEO datasets (GSE100675, GSE117423, GSE65626). (d) OTUB1 expression was examined in clinical samples. (e) OTUB1 protein was detected in clinical samples through IHC assay. (f – h) The correlation between the overall survival of glioma patients and OTUB1 expression was determined through online datasets (R2: Genomics Analysis and Visualization Platform)

OTUB1 expression was initially determined in glioma and adjacent tissues through the online datasets (GSE100675, GSE117423, and GSE65626), and found that OTUB1 expression displayed a remarkable increase in glioma tissues compared to that in the adjacent tissues Figure 1(a–c). Analyzing the clinical samples obtained a consistent result Figure 1(d,e). In addition, through analyzing different datasets, OTUB1 expression was found to be correlated with the poor overall survival of glioma patients Figure 1(f–h). Thus, these results suggest that OTUB1 might be an oncogene during glioma progression.

### Knockdown of OTUB1 reduces glioma stemness

**Figure 2.**
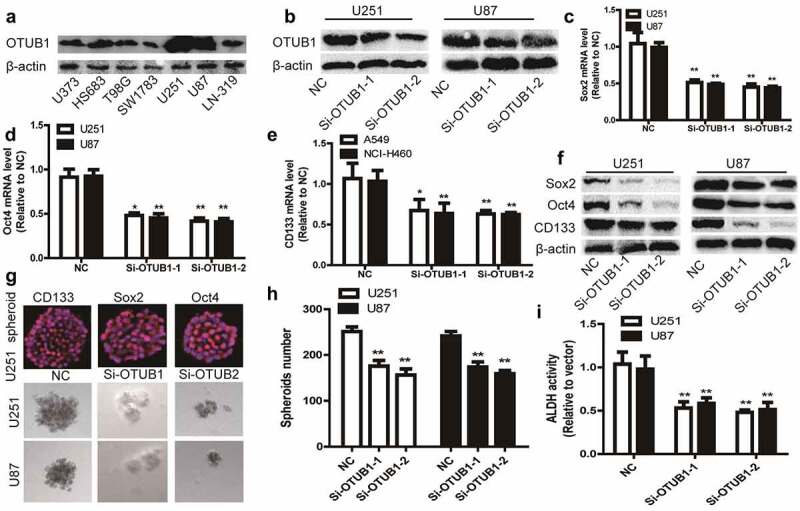
Knockdown of OTUB1 reduces glioma stemness. (a) Different glioma cell lines were subjected to detect OTUB1 protein level. (b) The knockdown efficiency of Si-OTUB1 was confirmed in U87 and U251 cells. (c – f) U87 and U251 cells with or without OTUB1 knockdown were subjected to determine stemness markers’ expression. (g and h) U87 and U251 cells with or without OTUB1 knockdown were subjected to evaluate tumor sphere number and size, and the expression of stemness markers using in situ immunostaining. (i) ALDH activity was measured in U251 and U87 cells with or without OTUB1 knockdown. n = 3, **P < 0.01 vs. Control

As CSCs positively engage in cancer occurrence and progression, we evaluated OTUB1 roles in glioma cell stemness. OTUB1 was knocked down in glioma cell lines U251 and U87 since the relatively higher expression of OTUB1 in these two cell lines and knockdown efficiency was confirmed Figure 2(a,b). It was found that the expression of stemness markers (Sox2, Oct4, and CD133) was decreased by OTUB1 knockdown Figure 2(c–f). Since the stemness of tumor cells can be characterized by the sphere-formation ability, we determined OTUB1 effects on the sphere-formation ability of glioma cells. As shown in Figure 2(g,h), OTUB1 knockdown significantly reduced the sphere-formation ability, as evident by the decrease of sphere number and size. Importantly, the expression of stemness markers (CD133, Oct4, and Sox2) was confirmed in spheroids using in situ immunostaining Figure 2(g). Furthermore, ALDH activity was attenuated by OTUB1 knockdown in glioma cells Figure 2(i). Therefore, these results prompt that OTUB1 contributes to glioma stemness.

### OTUB1 stabilizes SLC7A11 protein through directly interacting with it

**Figure 3.**
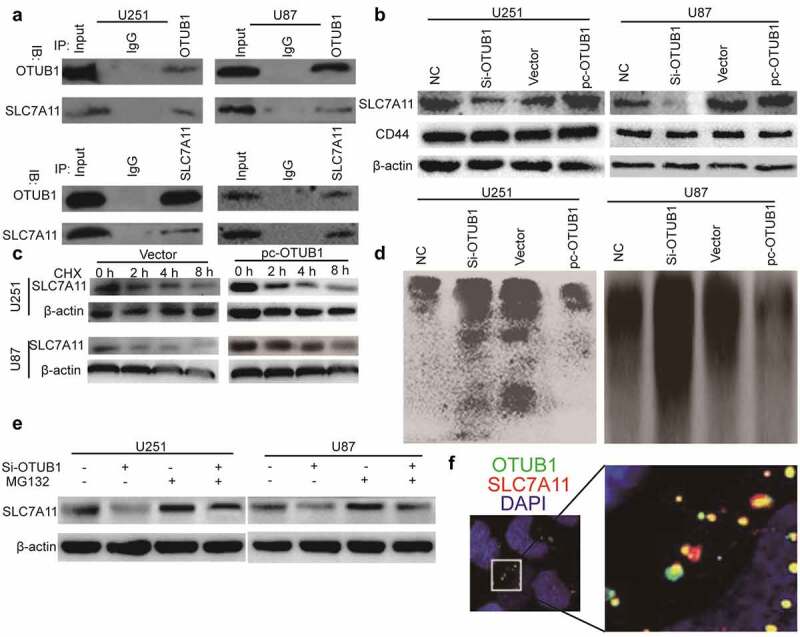
OTUB1 stabilizes SLC7A11 protein through directly interacting with it. (a) The OTUB1-SLC7A11 interaction was evaluated in U87 and U251 cells via co-IP assay. (b) U87 and U251 cells with OTUB1 knockdown or overexpression were subjected to detect SLC7A11 protein level. (c) U87 and U251 cells with OTUB1 overexpression were subjected to examine SLC7A11 protein level after being treated with CHX or not. (d) U87 and U251 cells with OTUB1 knockdown or overexpression were used to evaluate the ubiquitination of SLC7A11. (e) SLC7A11 protein level was determined in U87 and U251 cells with OTUB1 knockdown plus MG132 treatment or not. n = 3. (f) In situ staining for co-localization of OTUB1 and SLC7A11 in U87 cells

Given the known effect of OTUB1 on SLC7A11 and the critical role of SLC7A11 on ferroptosis [7], we next investigated whether the OTUB1-dependent effects of glioma cell stemness could be explained within such context. To evaluate the OTUB1-SLC7A11 interaction under physiological conditions, we performed co-IP assays in glioma cells. As shown in Figure 3(a), the endogenous SLC7A11 was co-precipitated by an OTUB1-specific antibody, while endogenous OTUB1 was co-precipitated by a SLC7A11-specific antibody. As OTUB1 belongs to be a kind of deubiquitinase, we further examined whether OTUB1 can regulate SLC7A11 protein stability through its deubiquitinase activity. As expected, SLC7A11 protein was markedly increased upon OTUB1 overexpression, while OTUB1 knockdown decreased SLC7A11 protein-level Figure 3(b). Notably, since CD44, another CSC marker, has been shown to be essential for the OTUB1-SLC7A11 interaction, we also detected the effects of OTUB1 on CD44 expression and found that OTUB1 had no efffect on CD44 protein expression Figure 3(b). Additionally, the half-life of SLC7A11 protein was significantly extended upon OTUB1 overexpression Figure 3(c). Consistently, the ubiquitinated level of SLC7A11 was increased by OTUB1 knockdown and reduced by OTUB1 overexpression, respectively Figure 3(d). Furthermore, the decreased level of SLC7A11 protein induced by OTUB1 knockdown was significantly restored upon treatment with MG132, a proteasome inhibitor Figure 3(e). Moreover, the co-localization of OTUB1 and SLC7A11 was confirmed in U87 cells using in-situ staining Figure 3(f). These data demonstrate that OTUB1 can stabilize SLC7A11 protein through suppressing SLC7A11 ubiquitination.

### OTUB1 knockdown triggers ferroptosis dependent on SLC7A11 expression

**Figure 4.**
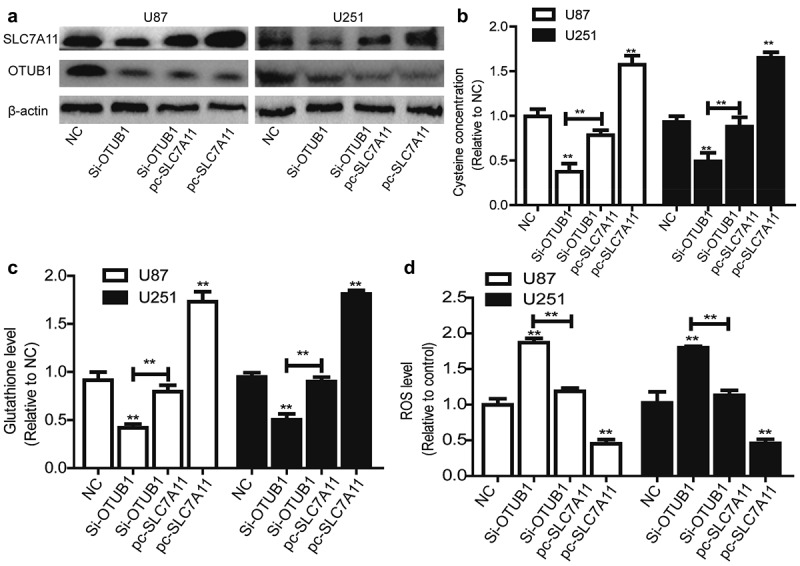
OTUB1 knockdown triggers ferroptosis dependent on SLC7A11 expression. (a) OTUB1 and SLC7A11 protein level was examined in U87 and U251 cells with OTUB1 knockdown as well as SLC7A11 overexpression or not. (b) The cysteine concentration was measured in U87 and U251 cells with OTUB1 knockdown as well as SLC7A11 overexpression or not. (c) Glutathione level was detected in U87 and U251 cells with OTUB1 knockdown as well as SLC7A11 overexpression or not. (d) ROS level was determined in U87 and U251 cells with OTUB1 knockdown as well as SLC7A11 overexpression or not. n = 3, **P < 0.01 vs. Control

Then we speculated that OTUB1 can regulate the ferroptotic process through SLC7A11 in glioma cells. SLC7A11 was overexpressed in glioma cells with OTUB1 knockdown. Western blot was conducted to confirm the co-expression efficiency and overexpression of SLC7A11 had no effect on OTUB1 expression Figure 4(a). As shown in Figure 4(b), OTUB1 knockdown significantly decreased cysteine level. Furthermore, glutathione level also was reduced in glioma cells with OTUB1 knockdown Figure 4(c). Consistently, OTUB1 knockdown increased ROS level in glioma cells Figure 4(d). Notably, all these above-mentioned effects led by OTUB1 knockdown were rescued by SLC7A11 overexpression Figure 4(b–d).

### SLC7A11 overexpression rescues the inhibitory effects of OTUB1 knockdown on glioma stemness

**Figure 5.**
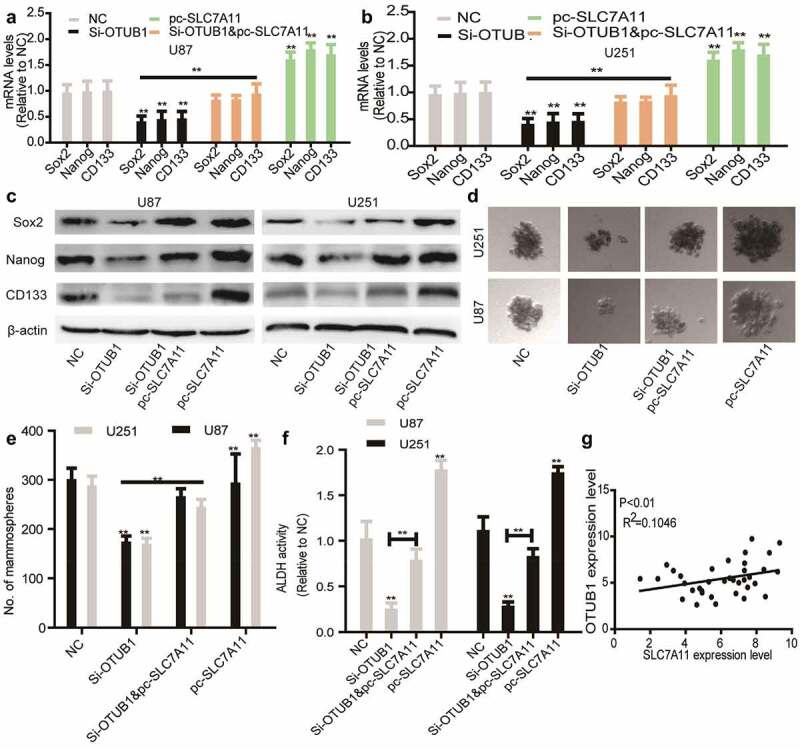
SLC7A11 overexpression rescues the inhibition of OTUB1 knockdown on the stemness of glioma cells. (a) Stemness markers’ mRNA levels were examined in U87 cells with OTUB1 knockdown plus SLC7A11 overexpression or not. (b) Stemness markers’ mRNA levels were detected in U251 cells with OTUB1 knockdown as well as SLC7A11 overexpression or not. (c) Stemness markers’ protein expression was measured in the cells depicted in (A) and (B). (d and e) The tumor sphere number and size were determined in the cells described in (A) and (B). (f) ALDH activity was tested in the cells described in (A) and (B). (g) The correlation between OTUB1 and SLC7A11 expression was evaluated in glioma tissues. n = 3, **P < 0.01 vs. Control

Finally, we wondered whether SLC7A11 is responsible for OTUB1 knockdown-induced effects on glioma stemness. As shown in Figure 5(a–c), SLC7A11 overexpression rescued the decreased expression of stemness markers led by OTUB1 knockdown. Additionally, OTUB1 knockdown-mediated inhibition of sphere-formation ability was abrogated by SLC7A11 overexpression Figure 5(d,e). Furthermore, OTUB1 knockdown-induced reduction of ALDH activity was partially reversed by SLC7A11 overexpression Figure 5(f). Notably, a positive correlation existed between the expression of OTUB1 and SLC7A11 in glioma tissues Figure 5(g). All in all, these results demonstrate that OTUB1 knockdown reduces glioma stemness dependent on SLC7A11 expression.

## Discussion

As a deubiquitinase, OTUB1 critically engaged in tumorigenesis and development, but its specific mechanisms contributing to glioma progression remain to be completely elucidated. Currently, we revealed that OTUB1 is overexpressed in glioma tissues through analyzing clinical samples and online datasets. Since CSC is a critical promoter for tumorigenesis and drug resistance, we examined the effects of OTUB1 on glioma stemness and found that OTUB1 positively regulates glioma cell stemness. However, OTUB1 effects on glioma cell viability are still unclear. This study firstly indicates that OTUB1 promotes the stemness of glioma cells. Notably, a previous work has demonstrated that OTUB1 knockdown attenuates the migration ability of glioma cells [12], this verifies OTUB1-induced promoting effects on glioma cell stemness as CSC contributes to cancer metastasis.

In order to explore the specific mechanisms contributing to OTUB1-mediated effects on glioma stemness, it is particularly important to identify new substrates with potential functions of OTUB1. SLC7A11 attracted our attention as it is identified as the substrate of OTUB1 in other tumors [24] and targeting SLC7A11 indeed can specifically kill CSCs in colorectal cancer [19]. Our results showed that OTUB1 positively regulates SLC7A11 protein level through an ubiquitinase-proteasome degradation system. Additionally, co-IP analysis indicated that OTUB1 directly interacted with SLC7A11 in glioma cells. Thus, this work identifies a novel OTUB1/SLC7A11 axis contributing to glioma cell stemness. Notably, a recent work indicated that SLC7A11 is also modulated by mTORC2-mediated phosphorylation [25]. We wonder whether this modification is involved in the OTUB1-SLC7A11 interaction in glioma cells. Furthermore, since MGMT and IDH1/2 have been established as the biomarkers for glioma treatment, we also detected the effects of OTUB1 on the protein expression of MGMT and IDH1/2 in glioma cells and found that OTUB1 knockdown decreased MGMT protein expression, while failed to affect IDH1/2 protein expression (Supplementary figure S1); however, the concrete mechanisms underlying OTUB1-mediated regulation on MGMT expression should be explored in the future.

Ferroptosis importantly engages in tumor progression and is becoming a new opportunity for cancer treatment [26]. Notably, the previous study has shown that sequestering iron in lysosomes could specifically kill CSC through triggering ferroptosis [27]. Further, works obtained the consistent result through using salinomycin-loaded gold nanoparticles [28]. In addition, the derivatives of this CSC-targeting salinomycin also can kill breast CSCs by the same mechanism of targeting lysosomal iron [29]. Furthermore, a recent study demonstrates that itraconazole specifically attenuates nasopharyngeal carcinoma stemness via triggering ferroptosis [30]. These data indicate that triggering ferroptosis might be a potential method to specifically kill CSCs. Thus, our results might provide another way triggering ferroptosis in CSC by promoting OTUB1 or suppressing SLC7A11. Specifically, numerous studies have indicated that targeting OTUB1 could induce myeloma cell apoptosis [31,32], and the anti-bacterial and anti-viral nanchangmycin has been shown to suppress OTUB1 and thus promote myeloma cell apoptosis [33], future efforts can be performed to determine the effects of nanchangmycin on glioma treatment.

## Conclusion

This firstly revealed the effects of OTUB1 on glioma stemness and provided a novel mechanism by which SLC7A11 protein, a critical suppressor of ferroptosis, is regulated in glioma cells. This study provides a potential target for glioma treatment by targeting the OTUB1/SLC7A11 axis.

## Supplementary Material

Supplemental Material

## Data Availability

All data generated or analyzed during this study are included in this published article and its supplementary data.

## Highlights

- A new critical role of OTUB1 in glioma cell stemness is proposed.
- The mechanisms of OTUB1 in glioma cell stemness are further studied.
- The mechanisms by which SLC7A11 protein was regulated are revealed in glioma.
- The novel mechanism provides new potential targets for glioma treatment.
