# Supplementary material for: The ubiquitin hydrolase OTUB1 promotes glioma cell stemness via suppressing ferroptosis through stabilizing SLC7A11 protein
Source: Bioengineered. 2021 Dec 19;12(2):12636–45. doi: 10.1080/21655979.2021.2011633 (PMC8810032; doi:10.1080/21655979.2021.2011633)
Supplement: Supplemental Material [file KBIE_A_2011633_SM2876.zip › supplementary/Supplementary Table 1.doc]

**Supplementary Table 1: Clinical pathological characteristics of the 36 glioma** patients

|  | **Frequency and treatment (Postoperative chemotherapy,%)** | **%** | **Low OTUB1** | **High OTUB1** | ***P* Value** |
| --- | --- | --- | --- | --- | --- |
| Age |  |  |  |  | 0.716 |
| Mean ± SD | 46.7 ± 6.2 |  | 48.2 ± 8.2 | 47.8 ± 9.2 |  |
| T grade of tumor |  |  |  |  | 0.018 |
| I | 10 (60.3%) | 27.8 | 5（50.0%） | 5（50.0%） |  |
| II | 8 (78.1%) | 22.2 | 4（50.0%） | 4（50.0%） |  |
| III | 8 (100%) | 22.2 | 2（25.0%） | 6（75.0%） |  |
| IV | 10 (100%) | 27.8 | 2（20.0%） | 8（80.0%） |  |
| Tumor size |  |  |  |  | 0.084 |
| ＜6 cm | 19 | 52.8 | 9(47.4%) | 10(52.6%) |  |
| ＞6 cm | 17 | 47.2 | 3(17.6%) | 14(82.4%) |  |
| Lymph node metastasis |  |  |  |  | 0.008 |
| Negative | 8 | 22.2 | 3(37.5%) | 5(62.5%) |  |
| Positive | 28 | 77.8 | 9(32.1%) | 19(67.9) |  |
